# Influence of cardiometabolic medications on abdominal aortic aneurysm growth in the UK Aneurysm Growth Study: metformin and angiotensin-converting enzyme inhibitors associated with slower aneurysm growth

**DOI:** 10.1093/bjs/znad375

**Published:** 2023-12-06

**Authors:** Corry Gellatly, Michael Sweeting, Atilla Emin, Emmanuel Katsogridakis, Sarah Finch, Athanasios Saratzis, Matthew J Bown, UKAGS Investigators and Collaborators, Furaha Asani, Manish Asiani, Jonathon Barber, Jamie Barwell, Sara Baker, Marcus Brooks, Neil Browning, Julie Chamberlain, Kundan Chandarana, Ian Chetter, Sohail Choksy, Caroline Clay, Alun Davies, Mark Dayer, Frank Dudbridge, Jonothan Earnshaw, Louis Fligelstone, Mark Gannon, Sarah Greatrex, Eric Grocott, Rajiv Pathak, Paul Hayes, Chris Imray, Shireen Kharodia, Sonja Khemiri, Tim Lees, Gabor Libertiny, Laki Liyanage, Charles McCollum, Shara McDonald, Colin Nice, Nik Oldham, Arun Pherwani, Lynda Pike, John Quarmby, Thomas Rix, Helen Rosendale, Nilesh J Samani, Rob Sayers, Cliff Shearman, Vince Smyth, Tim Sykes, William Tennant, John Thompson, Rao Vallabhaneni, Wafa Vayani, Syed W Yusuf

**Affiliations:** Department of Cardiovascular Sciences and NIHR Leicester Biomedical Research Centre, University of Leicester, Glenfield General Hospital, Leicester, UK; Department of Population Health Sciences, George Davies Centre, University of Leicester, Leicester, UK; Statistical Innovation, Oncology Biometrics, AstraZeneca, Cambridge, UK; Trauma & Orthopaedics, University Hospitals Coventry & Warwickshire NHS Trust, Coventry, UK; Department of Cardiovascular Sciences and NIHR Leicester Biomedical Research Centre, University of Leicester, Glenfield General Hospital, Leicester, UK; Department of Cardiovascular Sciences and NIHR Leicester Biomedical Research Centre, University of Leicester, Glenfield General Hospital, Leicester, UK; Department of Cardiovascular Sciences and NIHR Leicester Biomedical Research Centre, University of Leicester, Glenfield General Hospital, Leicester, UK; Department of Cardiovascular Sciences and NIHR Leicester Biomedical Research Centre, University of Leicester, Glenfield General Hospital, Leicester, UK

## Abstract

**Background:**

There is a clinical need for treatments that can slow or prevent the growth of an abdominal aortic aneurysm, not only to reduce the need for surgery, but to provide a means to treat those who cannot undergo surgery.

**Methods:**

Analysis of the UK Aneurysm Growth Study (UKAGS) prospective cohort was conducted to test for an association between cardiometabolic medications and the growth of an abdominal aortic aneurysm above 30 mm in diameter, using linear mixed-effect models.

**Results:**

A total of 3670 male participants with data available on abdominal aortic aneurysm growth, smoking status, co-morbidities, and medication history were included. The mean age at recruitment was 69.5 years, the median number of surveillance scans was 6, and the mean(s.e.) unadjusted abdominal aortic aneurysm growth rate was 1.75(0.03) mm/year. In a multivariate linear mixed-effect model, smoking (mean(s.e.) +0.305(0.07) mm/year, *P* = 0.00003) and antiplatelet use (mean(s.e.) +0.235(0.06) mm/year, *P* = 0.00018) were found to be associated with more rapid abdominal aortic aneurysm growth, whilst metformin was strongly associated with slower abdominal aortic aneurysm growth (mean(s.e.) −0.38(0.1) mm/year, *P* = 0.00019), as were angiotensin-converting enzyme inhibitors (mean(s.e.) −0.243(0.07) mm/year, *P* = 0.0004), angiotensin II receptor antagonists (mean(s.e.) −0.253(0.08) mm/year, *P* = 0.00255), and thiazides/related diuretics (mean(s.e.) −0.307(0.09) mm/year, *P* = 0.00078).

**Conclusion:**

The strong association of metformin with slower abdominal aortic aneurysm growth highlights the importance of the ongoing clinical trials assessing the effectiveness of metformin with regard to the prevention of abdominal aortic aneurysm growth and/or rupture. The association of angiotensin-converting enzyme inhibitors, angiotensin II receptor antagonists, and diuretics with slower abdominal aortic aneurysm growth points to the possibility that optimization of cardiovascular risk management as part of abdominal aortic aneurysm surveillance may have the secondary benefit of also reducing abdominal aortic aneurysm growth rates.

## Teaser

Abdominal aortic aneurysm growth rates were compared with smoking status, co-morbidities, and medication history in the UK Aneurysm Growth Study (UKAGS) prospective cohort. It was found that: smoking and antiplatelet use were associated with a more rapid abdominal aortic aneurysm growth rate; the drug metformin was strongly associated with a slower abdominal aortic aneurysm growth rate; and drugs in the angiotensin-converting enzyme inhibitor, angiotensin II receptor antagonist, and thiazide/related diuretic classes were also associated with a slower abdominal aortic aneurysm growth rate. These results suggest that common cardiometabolic medications may be useful therapeutic options to slow abdominal aortic aneurysm growth rates.

## Introduction

Screening for abdominal aortic aneurysm (AAA) in men above the age of 65 years, particularly in the UK, has resulted in large numbers of men in surveillance programmes with small AAAs (30–55 mm in diameter)^1,2^. There would be considerable benefits to identifying treatments that prevent AAA growth or slow it to the extent that the need for surgical intervention is greatly reduced, whilst also providing an alternative to surgical intervention in those unfit/unsuitable for surgery^3–5^.

Many commonly prescribed medications have received attention as potential treatments for AAA, but there is still no clear evidential basis for pharmacological treatment of the disease. Statins, for example, have been the subject of numerous observational studies and clinical trials, meta-analyses of which have reported conflicting results^6–10^. To date, there is insufficient evidence to state whether statins provide any protection against AAA.

Angiotensin-converting enzyme (ACE) inhibitors, angiotensin II receptor antagonists (ARBs), calcium-channel blockers, and antiplatelets have similarly been studied for their effect on AAA^11–16^, with inconclusive results. The beta-blocker propranolol was tested in an RCT and found to be poorly tolerated, with no significant effect on AAA growth rate^17^. The antiplatelet ticagrelor showed no effect on AAA growth in an RCT^18^.

An intriguing and consistent finding among AAA cohorts is that aneurysm growth is slower in diabetic individuals^4,13,19–23^; this has caused people to question whether the drug metformin (the most prescribed medication for type 2 diabetes) has the added effect of reducing AAA growth. In two separate studies, diabetic patients with AAA who were taking metformin showed a statistically significant reduction in aneurysm growth^24,25^. However, the numbers of patients in these studies was small (58 and 173 respectively).

There are no randomized trial data demonstrating any pharmacological treatment with a significant effect on AAA growth and there are conflicting results from observational studies. However, most studies have been small and underpowered, and an outcome such as AAA growth has considerable variability, which contributes to a high potential to miss true associations, especially if the effect size is small. Although there is tentative evidence for a number of medications, it is also possible that one or more commonly prescribed cardiometabolic medications may be impacting AAA growth, without any evidence already existing in the literature. The aim of this study was to identify any drugs or drug classes that potentially might be affecting AAA growth, either: through their general health outcome, such as reduction in cholesterol, blood pressure, or blood sugar; or via an unknown mechanism of action, which targets the AAA disease process. It did this by focusing on drugs within the cardiometabolic drug classes prescribed to participants in the study cohort.

The UK Aneurysm Growth Study (UKAGS) prospective cohort contains information on prescribed medications for a large number of participants who were not recruited on the basis of their medication status, offering the opportunity to observe associations between AAA growth and medication, unbiased by selection based on medication status. This study tested if any commonly prescribed medications are associated with AAA progression, using linear-mixed effect (LME) models, which are particularly suited to tests involving repeated measurements, dealing with variability in the number of measurements between subjects, correlations in data, and problems with multiple comparisons.

## Methods

### Study design

UKAGS is a UK national prospective cohort study, which has been conducted alongside the National Health Service (NHS) AAA Screening Programme (NAAASP) in England, but also accepts participants from Welsh, Scottish, and other regional independent screening and surveillance pathways. Ethical approval was granted by an NHS research ethics committee (REC reference: 09/H0406/119) and all patients provided written informed consent, in accordance with the Declaration of Helsinki.

### Patients and recruitment

In the UK, men are invited to attend for AAA screening in the year of their 65th birthday. This involves abdominal ultrasonography to measure maximum inner-to-inner aortic diameter. Men attending the AAA screening programmes in England, Wales, and Scotland were invited to join UKAGS, between 2011 and 2019.

### Questionnaires

Upon recruitment, participants completed a medical questionnaire. Data collected at baseline included patient biometrics, cardiovascular co-morbidities, smoking history, and quality-of-life metrics, using the Medical Outcomes Study Short Form 8 questionnaire format^26^. A complete list of current medications was also obtained via the questionnaire. Repeat questionnaires were posted out to be completed by the patients on a yearly basis, as previously described^27^.

### Abdominal aortic aneurysm scan data

Infrarenal AAA (inner-to-inner diameter) ultrasonographic measurements were obtained directly for England and Wales from NAAASP and three independent regional screening programmes and for Scotland and Wales from the Scottish and Welsh screening programmes. These data include repeated measurements taken at predetermined intervals depending on the AAA size. Institutions conducting AAA screening observe the guidance in the NHS clinical guidance document^28^. AAA growth estimates were calculated from the transverse plane measurements, where participants had two or more scans available from the point at which they were recruited to the study and where at least one scan showed a diameter of >= 30 mm, thereby signifying the presence of a small AAA.

### Smoking and co-morbidity data

The initial questionnaire responses enabled smoking status to be split into five categories (current smoker, ex-smoker, possible ex-smoker (indicated a smoking stop date contradicting other responses), never smoked, and unknown) and these could be modified to current smoker or ex-smoker by the response to the follow-up question ‘Have you smoked any form of tobacco in the last year?’. These categories were combined into a ‘current smoker/other smoking’ status binary variable for analysis. Individuals with unknown smoking history were excluded from all analyses.

Information on co-morbidities was taken from questionnaire responses and split into dichotomous variables, based on whether the individual had or had not reported the condition or clinical event. The participants were asked to respond ‘yes’ or ‘no’ to questions asking them whether they had ever had (baseline) or had in the last year (follow-up) a ‘heart attack’, ‘stroke’, ‘angina diagnosis’, ‘high blood pressure diagnosis’, and ‘diabetes diagnosis’. In the case of angina, high blood pressure, and diabetes, individuals were coded as ever having these conditions, whether they reported the condition in the baseline questionnaire or in a later one. In the initial coding of diabetes into a dichotomous variable, no distinction was made between type 1 and type 2 diabetes.

### Drug data

Lists of prescribed and non-prescribed medications and supplements, without dose data, were submitted by participants in each questionnaire. A process of data cleaning was carried out, whereby each medication listed was assigned to its formal drug name and drug class in accordance with the *British National Formulary*. An unbiased approach to inclusion of medications within the analyses was followed, restricted only on the basis that the medications were recognized treatments for cardiometabolic disease and that 30 or more participants had reported taking a medication within that class. This widely used minimum sample size for statistical tests was considered suitable for the study.

A sensitivity test was conducted to test whether individuals who reported taking a drug, but not in all questionnaires, needed to be treated as a separate group, from those who reported taking the drug in all questionnaires, for the purpose of statistical analysis.

### Statistical analyses

To test for differences in AAA growth rate, dependent on smoking status, co-morbidities, and medications, LME models were employed using the lme4 software package^29^ in R^30^. The LME approach deals with non-independence between successive aortic diameter measurements for each participant by using random effects. The models employed were correlated random intercept and slope models, in which the test variable was included as a fixed effect and interacted with time, to assess its effect on growth. Multivariate models were applied, in which all drugs, smoking, and co-morbidities were included within a single model as fixed effects and interacted with time, to account for co-prescription and to address possible confounding effects.

To assess whether inclusion of a test variable (for example smoking status, co-morbidity, or medication) improved the fit of the LME model (thereby indicating a potential influence of the test variable on AAA growth), an ANOVA F-test was conducted, using Satterthwaite’s method for calculation of degrees of freedom, via the lmerTest package in R. To account for multiple testing, the Bonferroni correction method was used to evaluate the statistical significance of the *P* values.

## Results

Some 3670 participants were included in the analyses, each with one or more completed questionnaire responses and two or more post-recruitment scans, with at least one scan >= 30 mm in diameter (*Table 1*).

**Table 1:** Clinical and demographic characteristics of cohort participants included and excluded from the analyses

Participants	AAA cases included*	AAA cases excluded*
*n*	3670	995
Sex	All male	All male
Mean age at recruitment (years)	69.5	69.0
Median number of surveillance scans	6	3 (*n* = 798†)
Mean AAA diameter at recruitment (mm)	37.3	35.6
Mean(s.e.) unadjusted AAA growth rate (mm/year)	1.75(0.03)	1.33(0.06)
Smoking status	Current smoker 658 Never smoked 480 Ex-smoker 2355 Possible ex-smoker 170 Unknown 7	Current smoker 75 Never smoked 62 Ex-smoker 295 Possible ex-smoker 19 Unknown 4 No questionnaire 540
Ethnicity	White 3464 Black Caribbean 2 Chinese 2 Indian 5 Pakistani 2 Unknown 195	White 431 Pakistani 1 Unknown 563
No medications reported in questionnaires	199	574

*The UK Aneurysm Growth Study (UKAGS) recruited non-AAA cases as controls, but these are not included in the table, as they were not eligible for the analysis in the first instance, given that it focused entirely on AAA growth. †A total of 197 participants had no post-recruitment scans. AAA, abdominal aortic aneurysm.

### Effect of smoking on abdominal aortic aneurysm growth

The UKAGS cohort is phenotypically similar to other AAA growth cohorts in terms of the effect of smoking, as evidenced by a highly significant faster AAA growth rate observed in current smokers compared with the ‘other’ smoking status group that included non-smokers and ex-smokers (+0.350 mm/year, *P* < 0.001, total number = 3663, unknown smoking status = 7), before adjusting for other factors.

### Co-morbidities associated with abdominal aortic aneurysm growth

LME models, controlling for smoking, but not taking into account medication history, showed that individuals who reported a diagnosis of high blood pressure had a significantly lower AAA growth rate than those who did not report a diagnosis of high blood pressure, after Bonferroni correction (−0.151 mm/year, *P* = 0.00769) (*Table 2*). The effect was stronger for those who reported a diagnosis of diabetes in any of their questionnaires, with a highly significant lower AAA growth rate than those without diabetes, after Bonferroni correction (−0.461 mm/year, *P* < 0.0002). No significant association with AAA growth was detected with respect to the other cardiovascular co-morbidities (stroke, heart attack, and angina) (*Table 2*).

**Table 2:** Analysis of aneurysm growth according to co-morbidities, not taking into account medication history

Variable	Number with/without	Growth rate (mm/year)	Co-morbidity effect(s.e.) (mm/year)*	*P*†
Heart attack	669/2994	1.816	−0.148(0.07)	0.03707
Stroke	290/3373	1.796	−0.078(0.1)	0.42221
Angina	422/3241	1.810	−0.158(0.09)	0.06752
High blood pressure	2041/1622	1.874	−0.151(0.06)	0.00769‡
Diabetes	612/3051	1.867	−0.461(0.07)	<0.00001§

Smoking status (current smoker/other) is included as a fixed-effect variable. *Adjusted only for smoking status and effect of smoking on growth rate. †*P* value calculated from an F-test with Satterthwaite’s approximation for degrees of freedom. Significance with Bonferroni correction at *n* = 5: ‡0.05/5 (0.01); and §0.001/5 (0.0002).

### Associations of cardiometabolic drugs with abdominal aortic aneurysm growth, by drug class

A sensitivity test was conducted to determine whether individuals who reported taking a drug in all questionnaires and those who reported taking a drug, but not in all questionnaires, should be included in single groups for analyses. It was found that there was a less than 0.3% change in growth rate estimation between these groups of individuals, with a less than 3.1% change in the standard error of the growth estimations. This was considered negligible and therefore all individuals who reported taking a drug were treated in single groups.

**Fig. 1:**
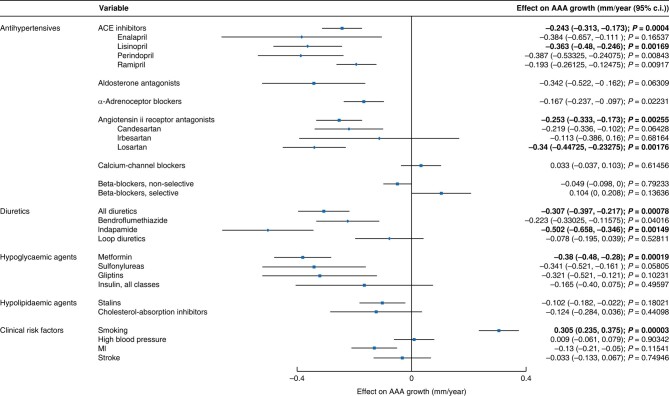
Effect size (mm/year) for drug classes tested for association with AAA growth, the component drugs for those significant classes, smoking, and cardiovascular co-morbidities, tested in multivariate LME model analyses Statistically significant results highlighted in bold. MI, Myocardial infarction; AAA, abdominal aortic aneurysm.

Among the drug classes eligible for analysis (*Table 3*), there is considerable co-prescription. For example, gliptins and sulfonylureas are almost entirely co-prescribed with metformin in the management of diabetes. To determine independent association with AAA growth and account for co-prescription, multivariate LME models were carried out, including all drug classes and co-morbidities within a single model, with the exception of diabetes, which was excluded due to collinearity with metformin. The models were a full pre-stepwise model (model 1) and a stepwise model (model 2) that involved exclusion of non-significant terms through a backward stepwise procedure (*Table S1* and *Fig. 1*). These models confirmed that smoking is highly significantly independently associated with more rapid AAA growth, after Bonferroni correction (model 1 +0.305 mm/year, *P* = 0.00003; and model 2 +0.305 mm/year, *P* = 0.00003), but did not confirm any independent effect of high blood pressure on AAA growth.

**Table 3:** Classes of drugs considered for analysis

Application/target	Included in the analysis	Excluded from the analysis*
Diabetes	Biguanides (metformin), gliptins, sulfonylureas, insulin (all classes)	Glucagon-like peptide-1 receptor agonists, meglitinides, sodium glucose co-transporter 2 inhibitors, thiazolidinediones
Antihypertensives	Angiotensin-converting enzyme inhibitors, angiotensin II receptor antagonists, antiplatelets, calcium-channel blockers	α- and β-adrenoceptor blockers, antihypertensives (centrally acting), peripheral vasodilators
Diuretics	Aldosterone antagonists, thiazides and related diuretics, loop diuretics	Potassium-sparing diuretics
Cholesterol lowering	Cholesterol-absorption inhibitors, α-adrenoceptor blockers, beta blockers (selective), beta-blockers (non-selective), statins	

^*^Excluded because there were fewer than 30 individuals who reported taking a drug in this class (in all questionnaires), who had sufficient scan data available.

ACE inhibitors (model 1 −0.243 mm/year, *P* = 0.00040; and model 2 −0.252 mm/year, *P* < 0.00004) and metformin (model 1 −0.38 mm/year, *P* = 0.00019; and model 2 −0.444 mm/year, *P* < 0.00001) exhibited a highly significant independent association with slower AAA growth, after Bonferroni correction. ARBs (model 2 −0.255 mm/year, *P* = 0.0012) and thiazides (model 1 −0.307 mm/year, *P* = 0.00078; and model 2 −0.286 mm/year, *P* = 0.00135) were also significantly independently associated with slower AAA growth.

Antiplatelets were highly significantly associated with more rapid AAA growth (model 1 +0.235 mm/year, *P* < 0.00018; and model 2 +0.193 mm/year, *P* = 0.00103).

### Associations of individual drugs with abdominal aortic aneurysm growth

To further investigate the results, the specific drugs within drug classes found to be significantly associated with AAA growth in the previous analysis were tested in multivariate models for their individual association with AAA growth. These were a full pre-stepwise model (model 3) and a stepwise model (model 4) that involved exclusion of non-significant terms through a backward stepwise procedure (*Table S2* and *Fig. 1*).

### Angiotensin-converting enzyme inhibitors

The only ACE inhibitor significantly associated with a reduction in AAA growth rate in the drug-specific multivariate models, using the stringent Bonferroni multiple testing correction, was lisinopril (model 3 −0.363 mm/year, *P* = 0.00169). No significant effect of the other ACE inhibitors (enalapril maleate, perindopril, and ramipril) was found, after correction for multiple testing. However, the direction and magnitude of effect for these drugs was comparable (toward slower AAA growth) and *P* values were close to the Bonferroni threshold for statistical significance (*Table S2* and *Fig. 1*).

### Angiotensin II receptor antagonists

The only ARB significantly associated with AAA growth, after correction for multiple testing, was losartan (model 3 −0.34 mm/year, *P* = 0.00176). No significant effect of the other ARBs (candesartan cilexetil and irbesartan) was found, though the direction and magnitude of the effect for these drugs (toward slower AAA growth) was comparable to that observed for the ARB class in the previous analysis (*Table S2* and *Fig. 1*).

### Thiazides and related diuretics

Bendroflumethiazide and indapamide were the only medications in this class with enough participants taking them to include in the drug-specific multivariate models. Only indapamide (model 3 −0.502 mm/year, *P* = 0.00149; and model 4 −0.482 mm/year, *P* = 0.00217) (*Table S2* and *Fig. 1*) reached Bonferroni statistical significance. The direction of effect was also toward slower AAA growth for bendroflumethiazide, though not significantly so.

### Antiplatelets

Aspirin and clopidogrel were the only medications in this class with enough participants taking them for these drugs to be included in the drug-specific multivariate models. They were not significantly associated with AAA growth, after correction for multiple testing.

To explore whether the association of antiplatelets with faster AAA growth at the class level might relate to greater use in individuals not taking other cardiovascular medications, the percentage of drug reports involving ACE inhibitors, ARBs, metformin, and thiazides was calculated, for individuals who reported taking antiplatelets. It is clear for all these classes of drugs that antiplatelet use is higher for those not taking a drug in that class. Those participants not taking antiplatelets were more likely to be taking ACE inhibitors (7.77% *versus* 7.26%), as well as ARBs (3.76% *versus* 3.03%), metformin (2.31% *versus* 2.07%), and thiazides (2.18% *versus* 1.97%).

### Diabetes, insulin, and metformin

Metformin was the only drug within the biguanide class, so there was no change in the number of individuals tested between the drug class and drug-specific multivariate models; the only difference was the other variables in the models. In the drug-specific multivariate models, it was again found that metformin was strongly associated with slower AAA growth, after correction for multiple testing (model 1 −0.539 mm/year, *P* < 0.00006; and model 2 −0.556 mm/year, *P* < 0.00006) (*Table S2*). Diabetes was excluded from these models, due to collinearity with metformin, so the interaction between diabetes, insulin, and metformin, using LME growth models controlling for smoking, was separately investigated.

Including all participants with smoking history, it was found that diabetes had a highly significant association with slower AAA growth (mean(s.e.) −0.461(0.07) mm/year, *P* < 0.001, number of cases/controls = 612/3051). The association was still highly significant when only excluding insulin users (mean(s.e.) −0.442(0.08) mm/year, *P* < 0.001, number of cases/controls = 568/3047). When excluding metformin users, the association was still significant, but at a much lower margin of error and with a lower effect size (mean(s.e.) −0.254(0.11) mm/year, *P* = 0.023, number of cases/controls = 244/3030).

## Discussion

AAA remains a significant disease, despite reports highlighting a decrease in prevalence^31–33^. In previous studies, smoking history and diabetes stand out as the two factors most consistently correlated with AAA growth (smoking with faster growth and diabetes with slower growth)^3,4,13,19–23^. These observations are confirmed by this study, which also revealed the association of a high blood pressure diagnosis with slower AAA growth. It is counterintuitive that diabetes and high blood pressure show an association with a reduced AAA growth rate, given that these are debilitating conditions, with adverse cardiovascular outcomes, but evidence from the medication histories reported by the UKAGS study participants points to the possibility that it may be the medications prescribed to treat these conditions that are affecting AAA growth.

In multivariate models correcting for co-morbidities and smoking, ACE inhibitors, ARBs, biguanides (metformin), and thiazide diuretics were associated, at a drug-class level, with a slower rate of AAA growth. Antiplatelets were associated with a faster rate of AAA growth. A breakdown of drugs within these classes subjected to the same analysis found lisinopril, losartan, metformin, and indapamide to be significantly associated with slower AAA growth. However, the individual drug analyses were carried out on smaller sample sizes, with a higher Bonferroni correction factor. The other ACE inhibitors, ARBs, diuretics, and antiplatelets that did not reach statistical significance in this analysis showed the same direction of effect. Arguably, this drug-specific analysis may give some indication of drug effectiveness, although is not sufficient to clearly distinguish the effectiveness of different drugs within classes.

The association of smoking with faster AAA growth was observed to be independent in multivariate models adjusting for the interactions with co-morbidities and medications. The association of high blood pressure with slower AAA growth was observed in a univariate model, but not in multivariate models, possibly indicating that the association is superficial and due to medications taken by individuals with high blood pressure.

An association of diabetes with reduced AAA growth rates is well established^4,23,34^, although the question remains whether it is diabetes itself that delays the pathogenesis of AAA or whether the effect is due to associated pharmacological treatment^24,25^. It has been shown that people with diabetes on treatment with agents other than metformin have similar AAA growth rates compared with those without diabetes, whilst those on metformin have a reduced rate of AAA rupture and need for intervention^35^, countering the idea that it is the diabetes itself that confers a protective effect, rather than treatment with metformin.

A limitation of the present study was the exclusion of diabetes from the multivariate models, due to collinearity with metformin. It has been hypothesized that the well-established association of diabetes with slower AAA growth is due to medication. However, this could not be confirmed by the present study. It was found that excluding metformin users from a univariate analysis of the effect of diabetes on AAA growth resulted in a large reduction in effect size and significance level, but the association was still statistically significant. This additional analysis did not control for other medications with a potential effect on AAA growth, for example ACE inhibitors, it only controlled for smoking, metformin, and insulin, and so was limited in its ability to determine true effects. It is clear, however, that individuals with diabetes who were taking metformin had the lowest AAA growth rates compared with the wider diabetes group.

The finding that ACE inhibitors, ARBs, and thiazides are associated with reduced AAA growth may suggest that the overall pharmacological management of cardiovascular risk factors is beneficial to AAA patients. ACE inhibitors have received much attention as a potential treatment for AAA, but there have been mixed results from retrospective cohort studies and clinical trials. An analysis of Canadian hospital admissions (sample of 15 326 patients, including 3379 cases) showed that prior use of ACE inhibitors was associated with a reduced risk of AAA rupture—a protective association that was not found for beta-blockers, calcium-channel blockers, alpha-blockers, ARBs, or thiazide diuretics^11^. In a similar finding from a nationwide Danish cohort study, which included 9441 AAA cases, ACE inhibitor prescription was associated with a reduced risk of surgery, whereas ARB prescription was not^12^. Other studies have either found no effect^13^ or a deleterious effect^14^ of ACE inhibitor prescription. The AARDVARK (Aortic Aneurysmal Regression of Dilation: Value of ACE-Inhibition on RisK) clinical trial found a slightly lower AAA growth rate over 2 years, but no significant effect of the ACE inhibitor perindopril or the calcium-channel blocker amlodipine^15^. The TEDY (Telmisartan in the Management of Abdominal Aortic Aneurysm) clinical trial also found no effect on AAA growth of the ARB telmisartan^16^. The present study differs from most previous studies in that it involves an extensive longitudinal component in the statistical analysis, whilst also allowing for analysis of ACE inhibitor and ARB use at the drug-class level. The finding of a protective association of ACE inhibitor and ARB use with AAA growth is not unexpected in the context of previous research and may point to a clinical benefit of blood pressure management in AAA patients.

The association of antiplatelet use with faster AAA growth may relate to an absence of treatment with other cardiometabolic medications, rather than harmful effects of the medications themselves, as it was found that participants were more likely to be taking antiplatelets if they were not taking ACE inhibitors, ARBs, metformin, and thiazides. However, this hypothesis was not confirmed statistically. Aside from smoking cessation, the results of this study suggest that AAA patients may benefit from treatment with diabetes and high blood pressure medications. However, these data are observational and subject to confounding. The question of whether prescription of such medications is of benefit to AAA patients, including those without a clinical indication for diabetes and high blood pressure, requires substantive clinical trial data to provide an answer.

A recent meta-analysis and a previous Cochrane review highlighted the absence of any single pharmacological agent that can be used to arrest or slow AAA growth^5,36^. These reviews, however, synthesized primary studies with considerable methodological limitations, including small study populations, retrospective design, and limited follow-up. The strength of the present study lies in it being a contemporaneous, large, real-world, and prospective analysis of data from the national screening programme, with a longitudinal component that is of sufficient duration for follow-up of patients diagnosed with AAAs.

A limitation of the study was patient reporting of medications via questionnaires, without dose data. Therefore, a recall bias leading to exclusion of pertinent medications cannot be discounted; neither can failure to comply with treatment—a problem common to most studies. Additionally, the possibility that a medication could have a dose-dependent effect, that has not been accounted for, should be considered. Moreover, individuals were coded as ever having diabetes, hypertension, or angina, whether they reported the condition in the baseline questionnaires or in later ones. It is possible that, in some cases, the participant was not suffering from the condition during the earlier part of their aorta scan history (or during the latter part, if the condition improved). However, it was decided to use this type of dichotomous approach, because it is likely that individuals were pre-hypertensive or pre-diabetic at baseline, if they later reported either condition, whilst the main cause of angina is atherosclerosis, which would likely have been developing at baseline if the participant later reported the condition. Exclusion of participants whose co-morbidity status changed midway through the study would have resulted in the loss of important data.

## Collaborators

**UKAGS Investigators and Collaborators**

Furaha Asani (University of Leicester, Leicester, UK); Manish Asiani (University of Leicester, Leicester, UK); Jonathon Barber (University of Leicester, Leicester, UK); Jamie Barwell (University Hospitals Plymouth NHS Trust, Plymouth, UK); Sara Baker (Royal Bournemouth Hospital, Bournemouth, UK); Marcus Brooks (North Bristol NHS Trust, Bristol, UK); Neil Browning (Ashford and St Peter's Hospitals NHS Foundation Trust, UK); Julie Chamberlain(University of Leicester, Leicester, UK);Kundan Chandarana (University of Leicester, Leicester, UK); Ian Chetter (University of Hull, Hull, UK); Sohail Choksy (Colchester Hospital, Colchester, UK); Caroline Clay (University of Leicester, Leicester, UK); Alun Davies (Imperial College, London, UK); Mark Dayer (Taunton Hospital, Taunton, UK); Frank Dudbridge (University of Leicester, Leicester, UK); Jonothan Earnshaw (Winfield Hospital, Gloucester, UK); Louis Fligelstone (HMT Sancta Maria, Swansea, UK); Mark Gannon (Queen Elizabeth Hospital, Birmingham, UK); Sarah Greatrex (University of Leicester, Leicester, UK);Eric Grocott (Nuffield Health Hereford Hospital, Hereford, UK); Rajiv Pathak (West Midlands Hospital, Birmingham, UK); Paul Hayes (University of Cambridge, Cambridge, UK); Chris Imray (University Hospitals Coventry and Warwickshire NHS Trust, UK); Shireen Kharodia (University of Leicester, Leicester, UK); Sonja Khemiri (University of Leicester, Leicester, UK);Tim Lees (Newcastle Hospitals NHS Foundation Trust, UK); Gabor Libertiny (Northampton General Hospital, Northampton, UK); Laki Liyanage (University of Leicester, Leicester, UK);Charles McCollum (University of Manchester, Manchester, UK); Shara McDonald (University of Leicester, Leicester, UK);Colin Nice (Freeman Hospital, Newcastle upon Tyne, UK);Nik Oldham (University of Leicester, Leicester, UK);Arun Pherwani (Royal Stoke University Hospital, Stoke-on-Trent, UK); Lynda Pike (Torbay Hospital, Torquay, UK); John Quarmby (University Hospitals Derby and Burton NHS Foundation Trust, UK); Thomas Rix (Kent & Canterbury Hospital, Canterbury, UK); Helen Rosendale (University of Leicester, Leicester, UK); Nilesh J Samani (University of Leicester, Leicester, UK); Rob Sayers (University of Leicester, Leicester, UK); Cliff Shearman (University of Southampton, Southampton, UK; Vince Smyth (Manchester Royal Infirmary, Manchester, UK); Tim Sykes (Shrewsbury and Telford Hospital, Shrewsbury, UK); William Tennant (Queen's Medical Centre, Nottingham, UK); John Thompson (University of Leicester, Leicester, UK); Rao Vallabhaneni (University of Liverpool, Liverpool, UK); Wafa Vayani (University of Leicester, Leicester, UK);Syed W Yusuf (University Hospitals Sussex NHS Foundation Trust, UK).

## Supplementary Material

znad375_Supplementary_Data

## Data Availability

Anonymized data underlying this article will be shared on reasonable request to the corresponding author.
