# Supplementary material for: Influence of cardiometabolic medications on abdominal aortic aneurysm growth in the UK Aneurysm Growth Study: metformin and angiotensin-converting enzyme inhibitors associated with slower aneurysm growth
Source: Br J Surg. 2023 Dec 6;111(1):znad375. doi: 10.1093/bjs/znad375 (PMC10763526; doi:10.1093/bjs/znad375)
Supplement: znad375_Supplementary_Data [file znad375_supplementary_data.docx]

**Influence of cardiometabolic medications on abdominal aortic aneurysm growth in the UK Aneurysm Growth Study: metformin and angiotensin-converting enzyme inhibitors associated with slower aneurysm growth**

Authors: Corry Gellatly^1^, Michael Sweeting^2,3^, Atilla Emin^4^, Emmanuel Katsogridakis^1^, Sarah Finch^1^, UKAGS Investigators and Collaborators, Athanasios Saratzis^1^, Matthew J. Bown^1^

^1^ Department of Cardiovascular Sciences and NIHR Leicester Biomedical Research Centre, University of Leicester, Glenfield General Hospital, Leicester, LE3 9QP, UK

^2^ Department of Population Health Sciences, George Davies Centre, University of Leicester, University Road, Leicester LE1 7RH

^3^ Statistical Innovation, Oncology Biometrics, AstraZeneca, Cambridge, UK

^4^ University Hospitals Coventry & Warwickshire NHS Trust, CV2 2DX, UK

**Corresponding author.**

Corry Gellatly^1^, [cg306@leicester.ac.uk](mailto:cg306@leicester.ac.uk). **ORCID ID:**  0000-0001-7458-2988

**Supplementary Materials - Index**

| **Supplementary Tables** |  |
| --- | --- |
| Table S1 | *page 2* |
| Table S2 | *page 4* |
|  |  |

Table S1. Analysis of aneurysm growth according to medication by class. Multivariable models including all drug classes, smoking and comorbidities. 342 participants reported no drugs in any of the classes tested. Model 1 includes all terms. Model 2 involves backwards stepwise procedure to remove terms with p-value >0.05 for fixed effect elimination and p>0.1 for random-effect elimination. Smoking status (current smoker / other) is included as a fixed effect variable. Significance with Bonferroni correction at n = 20: 0.05/20 (0.0025) = *, 0.01/20 (0.0005) = **, 0.001/20 (0.00005) = ***.

| **Drug class** | **n (3663 total)** | **Drug effect (s.e.) (mm/yr): multivariable model 1**^‡^ | **P-value multivariable model 1 ^¥^** | **Drug effect (s.e.) (mm/yr): multivariable model 2**^§^ | **P-value multivariable model 2 ^¥^** |
| --- | --- | --- | --- | --- | --- |
| ACE inhibitors | 1275 | -0.243 (0.07) | 0.00040* | -0.252 (0.06) | 0.00004*** |
| Aldosterone antagonists | 97 | -0.342 (0.18) | 0.06309 | -0.374 (0.17) | 0.03075 |
| Alpha-adrenoceptor blockers | 606 | -0.167 (0.07) | 0.02231 | -0.16 (0.07) | 0.02856 |
| ARBs | 569 | -0.253 (0.08) | 0.00255 | -0.255 (0.08) | 0.00120* |
| Antiplatelets | 2274 | 0.235 (0.06) | 0.00018*** | 0.193 (0.06) | 0.00103* |
| Beta blocking agents, non-selective | 75 | 0.049 (0.19) | 0.79233 | - | - |
| Beta blocking agents, selective | 1010 | 0.104 (0.07) | 0.13636 | - | - |
| Biguanides (metformin) | 389 | -0.38 (0.1) | 0.00019*** | -0.444 (0.1) | <0.00001*** |
| Calcium-channel blockers | 1159 | 0.033 (0.07) | 0.61456 | - | - |
| Cholesterol absorption inhibitors | 105 | -0.124 (0.16) | 0.44098 | - | - |
| Gliptins | 80 | -0.321 (0.2) | 0.10231 | - | - |
| Insulin, all classes | 48 | -0.165 (0.24) | 0.49597 | - | - |
| Loop diuretics | 217 | -0.078 (0.12) | 0.52811 | - | - |
| Statins | 2819 | -0.102 (0.08) | 0.18021 | - | - |
| Sulfonylureas | 106 | -0.341 (0.18) | 0.05805 | -0.398 (0.18) | 0.02507 |
| Thiazides and related diuretics | 377 | -0.307 (0.09) | 0.00078* | -0.286 (0.09) | 0.00135* |
| Smoking | 658 | 0.305 (0.07) | 0.00003*** | 0.305 (0.07) | 0.00003*** |
| High blood pressure | 2041 | 0.009 (0.07) | 0.90342 | - | - |
| Heart attack | 669 | -0.13 (0.08) | 0.11541 | - | - |
| Stroke | 290 | -0.033 (0.1) | 0.74946 | - | - |

¥ P-value calculated from an F-test with Satterthwaite’s approximation for degrees of freedom

‡ Co-adjusted for all other cardiovascular drug classes, smoking status, high blood pressure, heart attack, stroke, and effects of these covariates on growth rate. Diabetes excluded for collinearity with metformin.

§ Co-adjusted for smoking status, ACE inhibitors, Aldosterone antagonists, ARBs, Antiplatelets, Metformin, Gliptins, Sulfonylureas, Thiazides, and the following effects of covariates on growth rate (ACE inhibitors, Aldosterone antagonists, ARBs, Antiplatelets, Metformin, Sulfonylureas, Thiazides, smoking status). Diabetes excluded for collinearity with metformin.

Table S2. Analysis of aneurysm growth according to specific drugs. Multivariable models, including all relevant drugs, smoking and comorbidities. 795 participants reported none of the drugs tested. Model 3 includes all terms. Model 4 involves backwards stepwise procedure to remove terms with p-value >0.05 for fixed effect elimination and p>0.1 for random-effect elimination. Smoking status (current smoker / other) is included as a fixed effect variable. Significance with Bonferroni correction at n = 16: 0.05/16 (0.00313) = *, 0.01/16 (0.00063) = **, 0.001/16 (0.00006) = ***.

| **Drug** | **Drug class** | **N (3663 total)** | **Drug effect (s.e.) (mm/yr): multivariable model 3**^‡^ | **P-value: multivariable model 3 ^¥^** | **Drug effect (s.e.) (mm/yr): multivariable model 4**^§^ | **P-value: multivariable model 4 ^¥^** |
| --- | --- | --- | --- | --- | --- | --- |
| Enalapril Maleate | ACE inhibitors | 35 | -0.384 (0.28) | 0.16537 | - | - |
| Lisinopril | ACE inhibitors | 238 | -0.363 (0.12) | 0.00169* | -0.303 (0.11) | 0.00653 |
| Perindopril | ACE inhibitors | 139 | -0.387 (0.15) | 0.00843 | -0.363 (0.14) | 0.01127 |
| Ramipril | ACE inhibitors | 860 | -0.193 (0.07) | 0.00917 | -0.158 (0.07) | 0.01811 |
| Candesartan cilexetil | ARBs | 222 | -0.219 (0.12) | 0.06428 | - | - |
| Irbesartan | ARBs | 39 | -0.113 (0.28) | 0.68164 | - | - |
| Losartan Potassium | ARBs | 272 | -0.34 (0.11) | 0.00176* | -0.297 (0.1) | 0.00460 |
| Bendroflumethiazide | Thiazides | 256 | -0.223 (0.11) | 0.04016 | -0.222 (0.11) | 0.03669 |
| Indapamide | Thiazides | 110 | -0.502 (0.16) | 0.00149* | -0.482 (0.16) | 0.00217* |
| Aspirin | Antiplatelets | 1967 | 0.158 (0.06) | 0.00774 | - | - |
| Clopidogrel | Antiplatelets | 447 | 0.199 (0.09) | 0.02531 | - | - |
| Metformin | Biguanides | 389 | -0.539 (0.09) | <0.00001*** | -0.556 (0.09) | <0.00001*** |

¥ P-value calculated from an F-test with Satterthwaite’s approximation for degrees of freedom

‡ Co-adjusted for all other included cardiometabolic drugs, smoking status, high blood pressure, heart attack, stroke, and effects of these covariates on growth rate. Diabetes excluded for collinearity with metformin.

§ Co-adjusted for smoking status, Lisinopril, Perindopril, Ramipril, Losartan Potassium, Bendroflumethiazide, Indapamide, Aspirin, Clopidogrel, Metformin, and the following effects of covariates on growth rate (Lisinopril, Perindopril, Ramipril, Losartan Potassium, Bendroflumethiazide, Indapamide, Aspirin, Metformin, smoking status). Diabetes excluded for collinearity with metformin.
